# Machine, organism and language: a comparative epistemology of AI models

**DOI:** 10.1007/s00146-026-03094-7

**Published:** 2026-06-05

**Authors:** Matteo Pasquinelli

**Affiliations:** Department of Philosophy and Cultural Heritage, Ca’ Foscari University, Venice, Italy

**Keywords:** Epistemology, Organicism, Mechanicism, Cybernetics, Information theory, Linguistics, Structuralism, Artificial intelligence, Political economy

## Abstract

Inspired by Ernst Cassirer and Michel Foucault, this essay proposes a comparative epistemology of three paradigms central to the making of modern science, the humanities, and more recently AI: machine, organism, and language. These paradigms have influenced one another and recombined into complex analogies. Whereas the philosophy of science has often emphasised the organism-machine analogy from early modern mechanicism to cybernetics, this essay extends the inquiry to the language-machine analogy of late modernity, which runs from the telegraph and the Turing machine to information theory and Large Language Models. The rise of AI is thus framed as the confluence of these three paradigms, read not from an internalist perspective but from an externalist one, as mirrors of the social order. Against the dominant view of AI as a purely mathematical achievement or an imitation of biological intelligence, the essay argues that what AI systems automate are the relational structures sedimented in human cooperation, the division of labour, and culture at large—making AI, in effect, a model of the social manifold.

The history of thought is the history of its models. Classical mechanics, the organism, natural selection, the atomic nucleus or electronic field, the computer: such are some of the objects or systems which, first used to organize our understanding of the natural world, have then been called upon to illuminate human reality.

— Fredric Jameson, *The Prison-House of Language,* 1972: v.

## AI studies meet comparative epistemology

In Western modernity, the concepts of machine, organism, and language have played fundamental roles in the formation of science and the humanities, recombining into paradigms of increasing complexity at each turn of their evolution. They emerged as *abstract forms* and then as *scientific objects* claiming their own discipline. They developed into *philosophical paradigms*, *ideological worldviews, political mentalities,* and *cultural metaphors,* in which their role exceeded the scale of the originary observation and conception. To clarify the underlying paradigms of AI, this essay navigates the contested and shifting status of these *epistemic mediators* in the history of knowledge, in a way similar to previous observations made by Ernst Cassirer (1923) on *symbolic forms*, Ludwik Fleck (1935) on *thought styles,* and Lorraine Daston (2015) on *epistemic images*, yet resisting emulating illustrious predecessors such as Hegel (1807) on the figures of thought (*Gestalten des Denkens*). Summarily, one may say that machines inspired mechanicism, organisms organicism, and languages structuralism. Yet one has still to explain the circulation, contamination, and convergence of these paradigms, and the way they eventually contributed to the making of the technical mentality of AI.

The modern concepts of machine, organism, and language ramified across disciplines: for instance, in between engineering and biology, as well as in between linguistics and anthropology; and then, in the developments across cybernetics, information theory, and, ultimately, AI. In this latter case however, they have been rarely analysed together. As is well known, the belief that a machine could be designed as an organism was a key tenet of cybernetics, whilst information theory established the first material synthesis between language and machine, at the same time structuralism sought to describe kinship as a language. The philosophy of science and technology has often addressed the hybridization and contamination of the machine and organism paradigms, whilst leaving language aside. This essay attempts to interweave again the lineage of language into the one of the machine and organism, understanding this process as a political translation across disciplines.

Contemporary AI models, such as Large Language Models, can be seen as the convergence of the genealogy of machine, organism and language paradigms—a philosophical conurbation that emerged to encode, control and automate social relations into a new morphology. Although AI is commonly perceived as a mathematical achievement, it should instead be investigated through the paradigms that shaped its development and the worldviews these paradigms mediated. This essay, however, does not frame the paradigms of machine, organism and language as historical a priori: rather, as social mirrors diffracting other social mirrors, that is as ‘solutions to the problem of knowledge [that] are embedded within practical solutions to the problem of social order’ as Steven Shapin and Simon Schaffer (1985: 15) once registered in their study of seventeenth century natural philosophy.

Why is this exercise of political and epistemic translation, or *comparative epistemology,* necessary? Globalisation and late modernity scholarship have witnessed the rise of macro-paradigms at the crossroads of several disciplines that require increasingly complex research plans (see the Anthropocene debate, amongst others). In another season of intellectual complexity, at the peak of structuralism, Michel Foucault (1966/1994) attempted a similar synthesis of the modern episteme around the paradigms of life, labour and language. In these, one can recognise the traditions of natural sciences, political economy and linguistics respectively. Foucault’s main shortcoming was an excessive preoccupation with detecting *isomorphism,* rather than *causality,* in their developments: at the time, structuralism was advocating a focus on synchronic rather than diachronic configurations as a way to emancipate both philosophy and linguistics from the weight of historicism. Looking carefully, the categories of life, labour and language carry different historical and political weight and cannot be read on the same abstract continuum. Labour plays a fundamentally different role in historical dynamics than language. Although Foucault was concerned with rendering the two equal, philosophical inquiry today should acknowledge that the sciences of language rarely include labour in semiosis, whilst the sciences of labour rarely include language in production. Foucault’s concerns about isomorphism should be turned into concerns of translation, contamination and mutation.

As Marx (1858/1973) argued in the introduction to the *Grundrisse,* the modern category of labour is not a given, but a construction—the final articulation of deep historical processes of social abstraction. Indeed, due to its historical magnitude and complexity, the politics of labour cannot be left to the same *Wunderkammer* of fossil skeletons, as Foucault did. Foucault’s operation, nevertheless, can be read at the same level as Marx’s, as the intention to make the politics of labour a legitimate concern by positing labour as a scientific object. The aim of the present essay is similar, but not identical: it is about tracing the social and economic roots of key paradigms such as machine, organism and language, in order to illuminate them as mirrors and indices of the social order.

Foucault’s analogy of life and language failed to credit the last lecture that Ernst Cassirer (1945) gave in New York just before his death. His lecture consolidated the term ‘structuralism’ in English-speaking academia, framing it as a transformation of German organicism, which first rendered language as an (organic) system. Cassirer identified then a parallel between ‘structures’ in Saussure’s linguistics and Cuvier’s anatomy, whilst recapitulating his life-long project of a comparative epistemology of modern thought. In his *philosophy of symbolic forms*, Cassirer (1923, 1925, 1929) had already investigated science, art and mythology as emerging all from the same disposition of human civilisation toward abstraction. He defined, for this reason, the human as a *symbolic animal* (Cassirer 1944: 26). His inception of *Kulturphilosophie* legitimates the study of cultural forms beyond science and philosophy, predating the rise of cultural and media studies. Yet what his analysis (as well as Foucault’s) missed is an account of technology (Freudenthal 2017).

The present study advances a *comparative, historical and political epistemology of AI,* reading the genealogy of notions such as machine, organism and language that contributed to grounding AI research and design. It should be remembered that concepts often change their political polarity. For instance, at the end of the eighteenth century, Kant (1790) defined the concept of organism also to contrast the hegemony of theology on free will and the new social space that the new middle class was occupying. In the mid-twentieth century, cyberneticians (Wiener 1948) mobilised their ideal of the organism to foster the design of new machines and the agenda of military automation. Similarly, the machine was the form of social discipline in the industrial age (Marx 1867; Berg 1980), but after 1968, counter-culture and critical philosophy adopted it as a metaphor of empowerment against logocentrism: see, its role in French post-structuralism (Deleuze and Guattari 1972) as well as in the cyborg manifesto debate (Haraway 1985). Again, in information theory (Shannon 1948), the statistical analysis of language was key to improving the capacity of communication channels with no reference to the problem of meaning, whilst, in structuralist anthropology, the analysis of language was considered a point of access to the social unconscious (Lévi-Strauss 1949). Writing a historical epistemology of philosophical, scientific and technical notions means venturing into such contested territory.

**Fig. 1:**
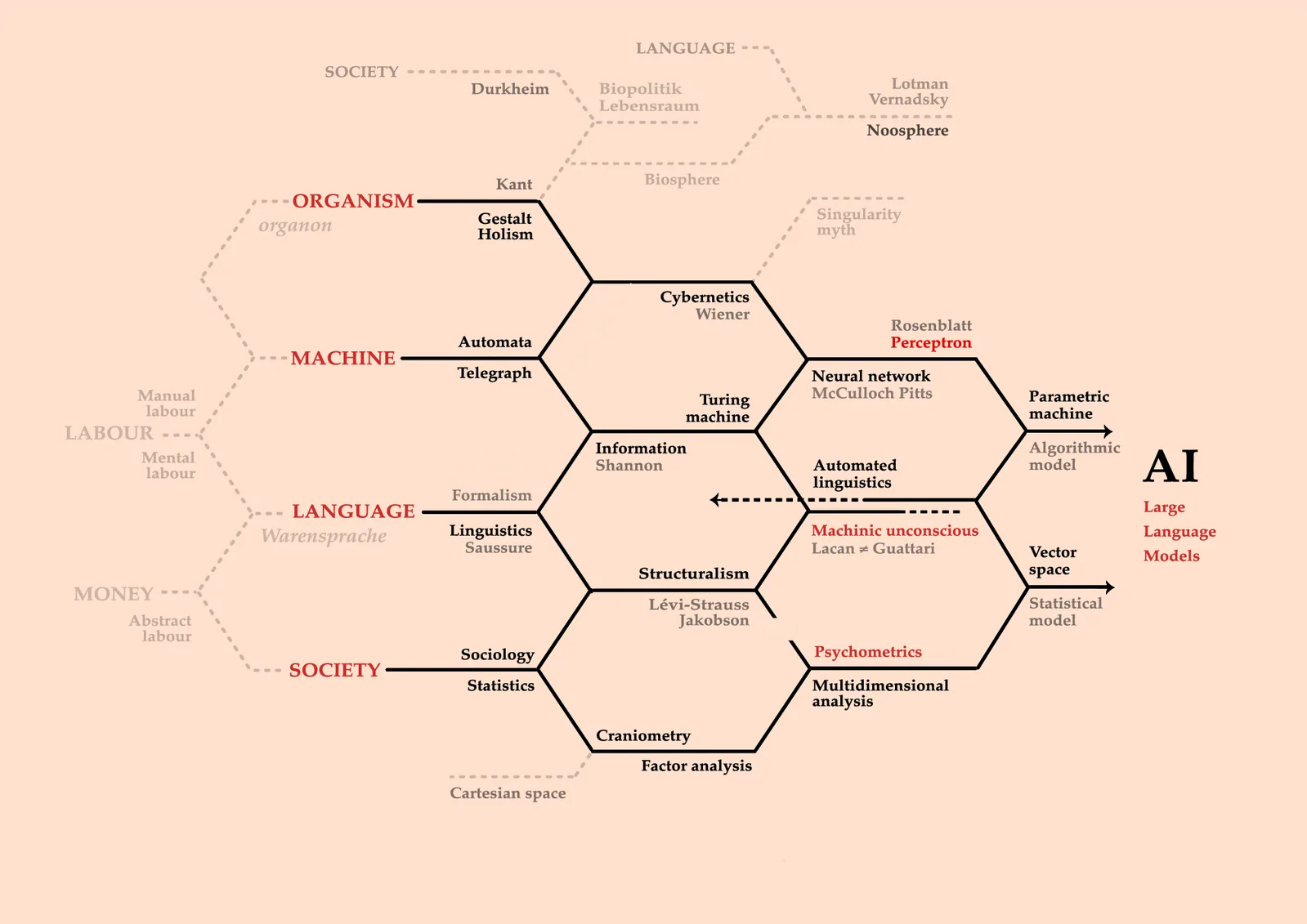
Diagram illustrating the genealogy of the paradigms of organism, machine, and language in relation to AI studies

The current essay deconstructs the making of AI as a composition of these three foundational paradigms that for historical reasons are presented in the following order: machine, organism and language. It questions two important analogies built upon these paradigms, namely the organism–machine and language–machine analogies, leaving aside, for reasons of space, the language–organism analogy (Fig. 1).

## The machine

The science of the living seems to be older than mechanics, because the organism appears to be natural and trans-historical, whereas the machine artificial and a recent achievement in human history. Yet this periodisation is misleading. Already in ancient Greece, the body was described through mechanical metaphors (Webster 2023). The modern term *organism* derives from the Greek term *organon, meaning* ‘tool’ of the body. These initial observations do not advocate for the primacy of a technological a priori over the experience of the world; rather, they acknowledge the historical a priori of social praxis over the abstractions of science. No scientific paradigm, especially a notion such as ‘intelligence’, should be received without assessing the mediation of cultural techniques and technical models in its genesis.

The machine has always been more than a material artefact: its technical materiality has generated philosophical paradigms, political mentalities and ideological worldviews, ultimately being rediscovered in its social genesis. Historians of science such as Franz Borkenau (1932) and Henryk Grossmann (1935) found a grounding for mechanical thinking in the economic sphere: they argued that the abstract diagrams of the division of labour and of the value form cast an epistemic influence on the abstract paradigms of modern sciences. The two authors, however, disagreed about the periodisation and context of this influence: Borkenau saw a direct influence of the abstraction of the division of labour on the abstractions of science, with Grossmann suggesting the key role of machines as mediators between labour and science. As feminist scholars (Merchant 1980; Federici 2004) have also highlighted, early modern Mechanical Philosophy and its mechanistic worldview developed in synchrony with a proto-mechanical discipline of the population, well before the machines of the industrial age established their own mechanical discipline. They argue that modern science (in particular, medicine) emerged to control women’s bodies and the social body in general, and not just to pursue a neutral scientific endeavour: in this way, at its inception, modern science mirrored and amplified an abstract mechanisation of the body and its social relations that was already in operation. The evidence of such mentality is today canonical. The vivid images of the treatise *De humani corporis fabrica* by Andreas Vesalius (1543) exemplify the early analytical view of modern medicine, whilst the ‘iatromechanics’ of animal bodies, as found in *De motu animalium* by Giovanni Borelli (1680), can be seen as a visionary forerunner of the division of labour and the ‘biomechanics’ of workshops and manufactures. These analytical and mechanical models were visions of the social order, and got gradually absorbed by modern philosophers such as Thomas Hobbes (1651) and René Descartes (1664), although in different ways. Federici (2004: 140) noted that Descartes mechanised the body to subordinate it to individual will, whilst Hobbes mechanised it to subordinate it to state power. In both cases, however, the body was redefined in preparation for the capitalist work discipline.

The early modern view of the machine remains analytical rather than transformative. It defined the mechanism as a disposition of elements with a high degree of freedom: once activated by an external force, the mechanism does not change its overall configuration, and can go back to the initial state. The mechanism’s elements are: independent in their function, replaceable with equivalent elements, and organisable in a different configuration (Canguilhem 1952/2022). In modern mechanics, time does not affect the operations of the mechanism: it is indifferent to temporal becoming and perfectly reversible. In the nineteenth century, the science of thermodynamics intervened to restructure this atemporal dimension and inscribed energy decay and transformation in the mechanism (Rabinbach 1992). Indeed, the machines of the industrial age are productive: they consume energy and transform matter, they turn raw materials into commodities. Whereas the machine of early modernity (e.g. loom) is analytical and reversible, the machine of late modernity is transformative and irreversible (e.g. engine).

The social status of the machine was widely debated in the so-called Machinery Question in early nineteenth century England (Berg 1980). It should be noted that the political economy of the industrial age recognised the primacy of social abstraction over mechanical abstraction. Against the belief that industrial innovation proceeds through the top-down application of science, key economists of different political orientations such as Adam Smith (1776), Charles Babbage (1832) and Karl Marx (1867) all maintained that the design of machinery emerges from the analysis of the division of labour from below. According to them, the machine design comes to imitate the diagram of a previous division of labour that has been consolidated into a productive routine. This principle (that can be termed a *labour theory of automation* in opposition to the mainstream *science theory of automation*) remains crucial also to understand the rise of computation and AI: it is now recognised that when Babbage conceived the first prototype of the digital computer known as the Difference Engine, he adopted Adam Smith’s principle of the division of labour to implement the automation of mental labour (Braverman 1974; Daston 1994, 2018; Schaffer 1994; Pasquinelli 2023).

It was Marx (1867), in particular, who systematised a theory of the machine as a part of a larger social antagonism. In his view, technological development was driven not only by the division of labour but also by workers’ resistance (strikes and sabotage), and importantly by capitalists’ drives to reorganise labour and extract relative surplus-value more efficiently. In Marx, a *labour theory* was dialectically entangled with a *standpoint theory* and *value theory of automation* (Pasquinelli, forthcoming). Such a political reading of the industrial machine pushed against the depoliticising and reductionist attitude of the industrial science of the time, particularly thermodynamics. The scientist Hermann von Helmholtz (1847) introduced the thermodynamic notion of *Arbeitskraft* to set on the same political continuum, without distinction, the energy performed by animals, workers and machines, suggesting a new metrics to measure and compare them (Rabinbach 1992; Pasquinelli 2022). Marx responded to such an equalisation with a political interpretation of *Arbeitskraft* that highlighted instead the labour–capital relation.

## The organism

The Medieval Latin noun *organisatio* and the later coinage *organismus* contain the Greek *organon,* which means tool and, metaphorically, ‘tool’ of a body (Webster 2023). Through modernity, both organisation and organism gradually came to refer to the disposition and assemblage of components that are oriented to a given purpose, one which mechanicism cannot fully represent. Tobias Cheung (2010: 155) has noted that ‘during the eighteenth century, the word ‘organism’ generally referred to a specific principle or form of order, often in opposition to the order of ‘mechanism,’ that could be applied to plants, animals or the entire world.’ Since then, the adjective ‘organic’ has become synonymous with the living, although in English it still maintains the meanings of ‘organised’, ‘systemic’, and ‘being part of’ with no necessary reference to life. As the previous part covered the making of the paradigm of the machine, this part focuses on the making of the organism in modernity in between a scientific object and philosophical concept (Wolfe 2010).

Immanuel Kant (1790) canonised the philosophical concept of organism in the *Kritik der Urteilskraft*, where he defined organic beings (*organische Wesen*) according to an internal teleological principle, rather than an external one. Kant’s elaboration must be read in its historical context, against the background of Newtonian mechanistic physics, on one hand, and, on the other, Christian theology, which maintained that the soul is inscribed in the living body by God alone. In polemics against both mechanicism and theology, Kant (1790: 555, §64) wrote that an organism is ‘cause and effect of itself’, an independent being in which form and finality coincide. As Evelyn Fox Keller (2008) noted, in this way, Kant provided the first definition of self-organisation that cast a long influence on natural sciences and even cybernetics and AI. The constituent parts of the organism are not independent (as in the machine) but inter-dependent and capable of reproducing each other (Kant 1790: 557, §65).

It should be noted that mechanicism posed no threat to a religious worldview: a machine is soulless and therefore compatible with God's role as prime mover. On the other hand, organicism entails an autonomy of the living, in opposition to the doctrine of the soul. It is perhaps no coincidence then that mechanicism was dominant in Catholic Europe, whilst organicism spread in Protestant Europe. The main lineage of organicism expands across the German tradition of *Naturphilosophie* (Humboldt 1845; Driesch 1908)*,* that sought an *organic unity* in all phenomena of the universe from crystals and living beings to social and cultural forms. Goethe’s (1790) quest for the *Urform* of living beings such as plants cast a lasting influence on German science that can be recorded up to the Berlin school of Gestalt psychology (Koffka 1935). German organicism situated the organism in a dialectical relation with the environment. Unlike the objective world (*Welt*), the environment (*Umwelt*) is the universe as experienced and projected from the subjective existence and point of view of a given organism. Haeckel (1866) coined the term ecology (*Ökologie*) precisely as the study of the interdependency between the organism and its environment. Similarly, Uexküll (1920b) defined the organism’s adaptation to its environment through an operative circuit (*Funktionskreis*): that is, a physiological feedback in which stimuli are continuously exchanged between the inner and outer world. The basic diagram of the cybernetic machine based on a feedback loop as a means of equilibrium with the environment can be traced back to Uexküll’s formalisation of the organism’s relation with the environment.

As Anne Harrington (1996: 3) recorded, the ‘call to Wholeness’ that animated German culture since the nineteenth century was a response to a dismembered country looking for unity and a reaction to the industrial crisis and alienation of urban masses. The movie *Metropolis* (Lang 1927) famously photographed the technophobic sentiment of the age. In addition, vitalist interpretations of the organism propelled far-right doctrines. The *Lebensphilosophie* contributed to biomorphic reactionary notions such as *Staatsbiologie* (Uexküll 1920a)*, Geopolitik* and *Biopolitik* (Kjellén 1917), and *Lebensraum* (Ratzel 1897), which Nazi Germany adopted to defend its right to expansion in analogy to the ‘natural’ growth of a life form. On the other hand, progressive readings of organicism can be found in the Gestalt school and in the neurology of Kurt Goldstein (1934/2000), Goldstein (1940). Goldstein’s concept of the mind as an organic unity implied not only capacity of self-organisation but also self-repair and self-regeneration after injury and trauma. Goldstein’s main work *Der Aufbau des Organismus* (1934/2000: literally, ‘The Structure of the Organism’) cast a deep influence on French philosophy and epistemology (Canguilhem 1943/1966; Merleau-Ponty 1942/1967) up to contemporary neurophilosophy (Metzinger 2004) and theories of neuroplasticity.

## The organism–machine analogy

The organism–machine analogy is a complex figure of thought which knows many variants in science, technology and philosophy. This part traces its progression in four historical positions without imposing a periodisation: iatromechanics, developmental mechanics, cybernetics, and organology. Iatromechanics was a branch of early modern medicine that considered the body of living beings as a mechanism, and described its parts as mechanical devices (Borelli 1680). Developmental mechanics, known as *Entwicklungsmechanik* in German, argued that embryos are subjected to the laws of mechanics and, therefore, ontogenesis can be experimentally modified following such laws (Roux 1895; Lenoir 1989). Cybernetics was grounded on a speculative postulate that machines can imitate the key principles of living beings, namely self-organisation, self-adaptation, self-generation and self-repair. It had the ambition of implementing the structure of the organism into a teleo-mechanism (Rosenblueth et al. 1943; Wiener 1948). Canguilhem (1952/2022) developed the concept of *organology* to stress that technology is an extension of living beings; therefore, the machine cannot be considered as an independent model for the organism, and rather the other way around is the case. These positions show that the history of the notions of machine and organism cannot be fully separated and that they constitute together a trans-systemic paradigm.

The organism–machine analogy is a field of contrasting worldviews. The concept of organism played a different role at the time of German idealism, Weimar culture, US cybernetics, French philosophy, and feminist debates. As already seen, for Kant, the organism was a philosophical concept to emancipate natural philosophy (and the bourgeoisie) from theology. During the Weimar Republic, the organism incarnated a cultural reaction to industrial alienation and a ’call to Wholeness’ against the fragmentation of the German nation (Harrington 1996). For cyberneticians, the organism grounded a modernist project of industrial automation against the background of new forms of social autonomy that animated Western society after WWII (Pasquinelli 2023). For Canguilhem, organology was a critical response to the new mechanistic worldview of cybernetics, at the same time as French structuralism was absorbing information theory as its episteme (Wolfe 2015). In the feminist debate of the 1970–80 s (Merchant 1980; Federici and Fortunati 1984; Federici 2004, et al.), the organism became an ideal subjectivity resisting modern sciences’ rule over women’s body, but it also represented an essentialist position to be abolished as in the programmatic myth of the cyborg (Haraway 1985).

In the development of AI, the organism–machine analogy played a key role that is different from the influence of the Turing Machine and its formalisation of language. As Keller (2008) noted, the automation of the principle of self-organisation was central to cybernetics and one can better understand early neural network research following this intuition over other principles of computation. Proceeding from holistic neurology, the idea of the brain as a system of neurons capable of self-organisation, self-adaptation and self-repair influenced Frank Rosenblatt (1958, 1962) in his invention of the neural network Perceptron, which is the prototype of the current deep learning architecture. Amongst other ideas, Rosenblatt attempted to implement in the Perceptron the basic Hebbian rule of neuroplasticity (Hebb 1949), usually paraphrased as ‘neurons that fire together, wire together.’ In the early 1960s, the US Army sponsored a series of conferences and research projects on the ‘principles of self-organisation’ that gave momentum to the idea of artificial neural networks as an alternative way to computation. These debates inspired also the conceptualisation of Arpanet, progenitor of the Internet, as a communication network that could reorganise itself in case of structural failures and external attacks, as biological neural networks do (Pasquinelli 2023: 149). The principle of self-organisation was in origin a morphological principle of adaptation rather than a logical one, but cyberneticians believed that discrete-state machines, such as digital computers, could nevertheless imitate it (Ashby 1947).

Because of its technomorphic drive to equate animals, humans and machines, cybernetics should be considered first a doctrine of social governance rather than a technical discipline (Wiener 1950 became aware of this). It is not difficult to see the cybernetic agenda to automate adaptation and learning as a technocratic view of society. Already in 1951, the political scientist Karl Deutsch (1951) described advanced societies as *self-organising learning networks,* an analogy in which the distinction between machine and society disappeared. Although quite reductionist, Deutsch’s view is prophetic of the current predicament in which the self-organising learning networks of AI are leading the automation of labour. Whilst cybernetics was keen to describe the machine as an organism or self-organising society, it was actually an ideological view implying society had to be organised as a machine. The principle of self-organisation that manifests itself in cybernetics and in current AI is not the imitation of a feature of the living, a principle of autonomy: rather, it is a principle of automation, that is of monopoly and control over the social bond.

## Language

Language became a scientific object of study only in late modernity. According to Foucault (1966/1994: 322): ‘From the nineteenth century, language […] became one object of knowledge among others, on the same level as living beings, wealth and value, and the history of events and men.’ Language was consolidated at the same time organism and labour were, respectively, consolidated in natural sciences and political economy: ‘The analysis of wealth is to political economy what general grammar is to philology and what natural history is to biology’ (Foucault 1966/1994: 182). However, according to Cassirer (1945), linguistics gradually emancipated itself from German organicism that saw language as an object of study analogous to a form of life. Emerging from philology, linguistics became akin to a natural science studying a form of life, and only later a human science studying a cultural form.

At the turn of the twentieth century, the philosophy of language gained momentum through its variants of positivism (Frege 1879; Carnap 1928; Wittgenstein 1922), which are not covered here. The positivist break in establishing language as an object of study in human sciences outside philosophy came from the publication of Ferdinand Saussure’s Cours de linguistique générale (1916) and of C. K. Ogden and I. A. Richards’ The Meaning of Meaning (1923). The former inaugurated a dyadic theory of the sign that grounded French structuralist linguistics, the latter a triadic theory of the sign that consolidated interpretative semiotics in the legacy of Charles Sanders Peirce (Eco 1976). Structuralism, then, became more hegemonic than interpretative semiotics because of its well-tempered blend of positivism, reductionism and relativism—and because of financial support by US military interests (Heller and McElhinny 2017).

Saussure’s (1916) dyadic theory is based on four main oppositions that seem to resolve language as an entity across the coordinates of a Cartesian plane. First, the historical development of language is divided into *synchronic* and *diachronic* dimensions. Second, language is then separated into *langue* (a given language as a system of rules) and *parole* (the act of speech in itself). Third, the act of speech is divided across *syntagmatic* and *paradigmatic* relations that represent language as a system of differences. Fourth, the sign is separated into *signifier* and *signified*. The key aspect of Saussure’s linguistics is that it conceived language as an abstract system of differences. Actually, it is the system as such that projects language:

In language, there are only differences. Even more important: a difference generally implies positive terms between which the difference is set up; but in language, there are only differences without positive terms. Whether we take the signified or the signifier, language has neither ideas nor sounds that existed before the linguistic system, but only conceptual and phonic differences that have issued from the system (Saussure 1916/2011: 149).

Jameson saw linguistics taking part in the early twentieth century crisis of sciences and contributing to the shift from substantialism to formalism, in a similar way in which Cassirer (1910) recorded the shift from the concept of substance to function in physics. Jameson (1972: 14, 35) did not see a continuity between organicism and structuralism, rather a break with the organic obsession. Going beyond organicism, Saussure's structuralism is a theoretical turn in which objects become the effect of an extended field of relations—that is, of the system.

The linguist Roman Jakobson (1929/2023), (1961a), 1961b) played a central role in consolidating structuralism and exporting its worldview to other disciplines, above all anthropology (Lévi-Strauss 1949). Structuralism took language as the foundational homology for all social structures—from kinship systems to mythologies, from design artefacts to ideologies (Dosse 1997, 1998). It sought to politicise these ‘structures’ and to reinterpret political economy (including Marxism) through a linguistic lens: capital itself was understood as a ‘semiotic operator.’ Ultimately, structuralism was contested by post-structuralism, which advanced a philosophy of subjectivity against its logocentrism. ‘Structures do not take to the streets’, declared a well-known graffiti from 1968. Post-structuralism exposed structuralism’s limitations, yet something was lost in the antagonism between them. Because of its harsh critique of linguistic abstraction, post-structuralism (see especially Deleuze and Guattari 1972) was left ill-equipped to confront the automation of language in late capitalism.

According to Jameson, structuralism was not a neutral scientific achievement, but rather a projection of the political composition of its days. The adoption of language as a model across disciplines other than linguistics had to be found not in scientific validity or technological progress, but:

in the concrete character of the social life of the so-called advanced countries today, which offer the spectacle of a world from which nature as such has been eliminated, a world saturated with messages and information, whose intricate commodity network may be seen as the very prototype of a system of signs. There is therefore a profound consonance between linguistics as a method and that systematized and disembodied nightmare which is our culture today. (Jameson 1972: viii)

Nevertheless, in the mediation of this ‘disembodied nightmare’, which is another name for commodity fetishism, one should not overlook the role of technology. In the years of structuralism, for instance, Lacan did not overlook this aspect and described the regime of the Symbolic (i.e. language) as a common ground between the unconscious and cybernetic machines.

We are very well aware that this machine doesn't think. We made the machine, and it thinks what it has been told to think. But if the machine doesn't think, it is obvious that we don’t think either when we are performing an operation. We follow the very same procedures as the machine. […] Through cybernetics, the symbol is embodied in an apparatus—with which it is not to be confused, the apparatus being just its support. And it is embodied in it in a literally trans-subjective way. (Lacan 1955/1988: 304)

Structuralism and cybernetics emerged almost simultaneously, but the success of the former was also due to the influence of the latter on French thought (Lafontaine 2007; Liu 2010; Geoghegan 2023). Behind structuralism was not only Saussure’s linguistics, but also the formalisation of language enforced by early information technologies such as telegraphy. Jakobson presented at the Fifth Macy Conference on cybernetics and linguistics in 1948 (Van de Walle 2008), and later recognised that:

‘There appear indeed striking coincidences and convergences between the latest stages of linguistic analysis and the approach to language in the mathematical theory of communication. […] The dichotomous principle underlying the whole system of distinctive features in language was gradually disclosed by linguistics and found corroboration in the binary digits (or to use the popular portmanteau, bits) employed as a unit of measurement by the communication engineers’ (Jakobson 1961b: 245).

His modernism and, at the same time, limit (as per Lacan) was to think that linguistics and information theory share the same principles. Structuralism and cybernetics surely shared a common root but this has to be identified in the controversial language–machine analogy, rather than in language per se.

## The language–machine analogy

As much as the organism–machine analogy is key for understanding the modern episteme until cybernetics, the language–machine analogy plays a similar role for the twentieth century and the age of AI. The analogy is about the possibility that a machine can emulate and automate the generative structure of language—the interplay of ‘information, mechanism, and meaning’ (MacKay 1969). This issue can be historically illustrated by looking at the telegraph as an *epistemic mediator* between the domains of machine and language in late modernity (Wise 1988). Telegraphy performed a material and heuristic *formalisation of language* into bits of information that influenced, with the help of mathematics, the rise of both information theory and structural linguistics. In the history of science, the telegraph represented a watershed innovation: it de facto implemented a formalisation of language into a mathematical code (i.e. Morse code), often under-recognised as a forerunner of the binary code of the digital age. In different intellectual moments, the telegraph played the role of *model machine* for sensation, cognition and computation. In nineteenth century Berlin, scientists Emil du Bois-Reymond and Hermann von Helmholtz (Hoffmann 2003) took the telegraph as a model for the physiology of the nervous system (Otis 2001). British mathematician Alan Turing (1936, 1950) adopted the telegraph as a model of the Turing Machine, and implicitly also in the ‘imitation game’ known as the Turing Test. US mathematician Claude Shannon (1938, 1948) inaugurated information theory as a quantification of language in telegraphy to solve problems of compression, encryption and transmission along communication channels. After Shannon, cyberneticians Warren McCulloch and Walter Pitts (1943, 1947) adopted the telegraph as the model to formalise biological neurons into artificial neurons. At the 1948 Hixon Symposium at the California Institute of Technology, McCulloch insisted on conceiving ‘neurons as telegraphic relays’ (Jeffress 1951:45).

Looking attentively at the history of cybernetics and information theory, it must be noted that two paradigms of information co-existed through two models of machine, which engendered further hybrids, such as the current form of AI. The coupling of the paradigms of machine and organism was the concern of Wiener (1948). The coupling of the paradigms of machine and language was the concern of Shannon (1948). The former adopted the model of information from organicist biology (Uexküll 1920b), the latter from military telegraphy and signal intelligence. Wiener maintained a model of information that was substantially analogue, concerned with control and adaptation, whilst Shannon advanced a discrete one, concerned with encoding, bandwidth and transmission. It is the latter paradigm of *linear information* (i.e. information codified in a linear medium such as written language) that became hegemonic and grounded the computer age. However, the current form of AI emerged from the former paradigm of *self-organising information*. More precisely, one should see the invention of artificial neural networks such as the Perceptron (Rosenblatt 1958, 1962) as the confluence of the two paradigms of organism–machine and language–machine. The confluence of these two paradigms can be easily detected also in McCulloch and Pitts (1943, 1947), when they introduced the idea of artificial neuron. The artificial neuron (McCulloch and Pitts 1943) was envisioned to encode Boolean logic with the later ambition to automate the recognition of visual patterns (McCulloch and Pitts 1947). Today, deep neural networks, such as LLMs, are computing networks made of trillions of parameters (also known as *parametrised machines*) that adjust and self-organise their weights in order to compute a statistical model of large data repositories.

Looking at the trajectory of computation over the last century, the formalisation of language is found both at its inception—in telegraphy and information theory—and at its culmination in the planetary hegemony of LLMs. Shannon (1948) founded information theory to quantify the ‘intelligence’ or interpretability of a signal in telegraphy, drawing on earlier work from Andrey Markov, who analysed the first 20,000 letters of Pushkin’s *Eugene Onegin*. Shannon defined ‘information’ as a statistical measure of token frequency in printed English. The intuition of digital information, in fact, is not simply about the discretisation of language into a binary code, as in telegraphy: it is an index of the predictability of its letters into sequences and compounds. It is often said that information theory is not concerned with content (*semantics*), but with reducing language to its grammatical structure (*syntax*). Like Markov, Shannon sought to demonstrate that a natural language is statistically dependent: therefore, any text can be measured, computed and transmitted as a stable message across a noisy channel. In time, computation grounded the large statistical models of AI, which came to analyse and automate increasingly complex linguistic artefacts, inspiring a new phase in linguistics, that is the *automation of linguistics* (Léon 2015/2021).

Thus, LLMs revive the old structuralist hypothesis that language is a vast system of dichotomic differences which now can be measured accurately as complex positions in a vector space. Old structuralism turned into neo-structuralism (Guariento 2026): whereas the former encoded meaning as a binary opposition or dichotomy in a low-dimensional space, the latter measures meaning as cosine similarity in a multi-dimensional vector space of greater flexibility. The performance of deep learning neural networks (such as Transformer models, e.g. ChatGPT) has given new impetus to the ergodic theory in linguistics according to which AI models would demonstrate the computability of human language as such. Already Shannon (1948) had treated language as a ‘stochastic process’ and noted that an English corpus behaves like a ‘stationary ergodic source’ for the purposes of prediction. By training on massive textual corpora (treated as samples from an underlying stationary ergodic linguistic source), contemporary LLMs behave as if the long-run frequencies of words and constructions can approximate the full generative rules of language. The Italian semiotician Umberto Eco once warned that this process is algorithmically impossible, because language is a creative and living process, not just the imitation of the past. One of the entities that LLMs struggle to decode and encode is a new poetic metaphor: ‘No algorithm exists for the metaphor, nor can a metaphor be produced by means of a computer’s precise instructions, no matter what the volume of organized information to be fed in’ (Eco 1986: 127).

## The historical synthesis of machine, organism and language

AI’s position in the history of science and technology can be understood as the confluence and synthesis of the three paradigms of machine, organism and language. From this perspective, an AI system has to be understood as a machine that reorganises its own parameters like an organism adapting to its environment in order to model the language of social artefacts. This movement of self-organisation is not simply a technical feature: it reflects the social genesis of technology in general, that is an emergent process in which machines internalise the relational structures of collective practices—structures that include human cooperation, the division of labour across society, and the collective dynamics of language and culture (one should also define language, by the way, as a process of self-organisation). Cybernetics had already framed self-organisation as the capacity of a system to internalise and reproduce the relational structures present in its environment. AI follows this logic at scale: today it takes the form of a vast parametrised machine that maps the dense networks of relations sedimented in data and cultural artefacts. Texts, images and sounds are not isolated entities, but matrices generated by collective practices; semiotically, they are diagrams of social relations. As a matter of fact, what AI automates is not biological intelligence, but the relational structures historically produced by human cooperation (Pasquinelli 2023).

These insights were familiar to cyberneticians such as Karl Deutsch, who in 1951 authored the programmatic essay ‘Mechanism, Organism, and Society’, as an attempt to trace analogous models in natural and social sciences. Deutsch framed the history of thought as the evolution of mechanicism, organicism and historicism culminating in cybernetics. He believed that cybernetics helped to recognise profound ‘analogies […] between communication channels or control processes in machines, nerve systems, and human societies’ and he identified them in the principle of ‘self-modifying communications network or learning net’ (Deutsch 1951: 240). Deutsch’s description of both the project of cybernetics and the ontology of society as a self-organising learning network reads as distinctly technocratic, yet it is quite prophetic of the current predicament of AI. In a way similar to the labour theory of automation and technological development seen above, Deutsch observed that the increasing division and mechanisation of labour had prepared the way for the division and mechanisation of mental labour in cybernetics (and AI):

Just as the division of manual labor between different pairs of human hands preceded the division of labor between human hands and power-driven mechanisms, so in a sense the increasing division of intellectual labor between different human minds has preceded today's growing divisions of labor between human minds and an ever-growing array of electronic or other communications, calculating, and control equipment. (Deutsch 1951: 239)

What cybernetics depicted as a self-organising learning network is a machination of the same relational structures that constitute linguistic cooperation and social life. Seen from this angle, the machine, organism and language genealogy acquires a sharper meaning and points once again to the social synthesis that this essay attempts to illuminate: AI is a model of the social manifold.

## Questioning language machines

In post-war France, Canguilhem addressed the organism–machine analogy to contest the rise of cybernetics’ mechanist paradigm in science and philosophy. He criticised the analogy because ‘life tolerates monstrosities’, whereas ‘there is no machine monster […] no mechanical pathology’—meaning that cybernetics could never express the *abnormality* of life (Canguilhem 1952/2022: 90). It appears as if Canguilhem defended the plasticity of the Organism, its capacity of exceeding the norm against the rigidity of the Machine. However, his argument was more profound. Revisiting the controversy between Franz Borkenau (1932) and Henryk Grossmann (1935), Canguilhem traced the rise of the mechanist paradigm to its disciplinary and economic roots, that is the exploitation of labour power and of life in general. Canguilhem (1952/2022: 84) in fact argued that: ‘The theoretical mechanisation of life and the technical utilization of the animal are inseparable.’ Today, a similar observation could be made regarding the language–machine analogy, as AI starts dominating labour automation, cultural production, and even the humanities. In the age of AI, one could argue that the theoretical mechanisation of language and the technical exploitation of culture are deeply imbricated.

To address the disputed language–machine analogy, I suggest looking at the constitutive role of language in the sphere of labour. This requires reversing a trajectory of analysis that has become habitual in critical theory. Rather than simply recording how the machine alienates language, one should recognise that language has contributed to the machine design since its inception. As Hegel (1805, 1807) argued in the Jena lectures and in the *Phenomenology of Spirit*, it is the abstraction of human activity—labour as much as language—that creates the machine. Labour and language are machinic in their own right before the machine comes to alienate them, pointing thereby to a dimension that exceeds mechanisation. To deconstruct the final segment of the machine–organism–language genealogy, this concluding part moves from Hegel’s insight and briefly highlights other moments in philosophy and political economy in which language is discussed in its economic role.

Long before digital computers came to model language, language once happened to be conceptualised as a machine. In the circles of Soviet avant-garde art, formalists Viktor Shklovsky and Boris Tomashevsky framed the literary text as a mechanical device (Steiner 1984). Their poetics must be read against the backdrop of Soviet drives toward industrial automation and labour politics: in this context, it would not be difficult to envision a text as a *productive machine*, as a structure of visible and invisible relations aiming at causing an effect upon a collective audience. One could also easily define the literary text as a machine that emerges from the division of labour and language. A decade later, the US linguist Leonard Bloomfield (1933: 24) argued that ‘the division of labour, and, with it, the whole working of human society, is due to language’, hinting that language is part of the inner constitution of labour. Language works, language produces, and the product of this work, whether material or immaterial artefacts, shares the same fabric of language. Texts and books are alive, they respond to a large network of social interactions, to the vast social abstraction of language. In short, before digital computers imposed their *machine language,* and well before AI took over with its *language machines*, Soviet formalists had already envisioned the machine of language. They conceived linguistic artefacts as elements of a larger productive mechanism, much as one could view LLMs today, but with a crucial difference. Their poetics was not productivist, but rather *alienist*, seeking through art the effect of *ostranenie,* or estrangement (Shklovsky 1917/1991).

In the circles of French structuralism, the main successor of Russian formalism, the analysis of language, on the other hand, began gravitating around the sphere of circulation rather than production. Saussure (1916/2011: 79) famously wrote that: ‘[In linguistics] as in political economy we are confronted with the notion of value; both sciences are concerned with a system for equating things of different orders—labor and wages in one and a signified and signifier in the other.’ Saussure’s analogy between linguistic sign and economic value captivated numerous structuralists and Marxists (Barthes 1964, Lefebvre 1966, Goux 1973; and recently Tomšič 2015, Khatib 2017, Ricci 2024). Marx (1867) himself spoke of a *Warensprache,* a language that commodities would speak to one another, and this image persuaded many to recognise language more and more often in the sphere of circulation, within the *value form*, rather than in production, within the *labour form*, although Marx himself warned:

To compare money with language is not less erroneous. Language does not transform ideas, so that the peculiarity of ideas is dissolved and their social character runs alongside them as a separate entity, like prices alongside commodities. Ideas do not exist separately from language. Ideas which have first to be translated out of their mother tongue into a foreign language in order to circulate, in order to become exchangeable, offer a somewhat better analogy; but the analogy then lies not in language, but in the foreignness of language. (Marx 1858/1973: 162)

In the years of structuralism and Foucault’s attempts at a comparative epistemology of labour and language, the Italian philosopher Ferruccio Rossi-Landi (1968, 1977) provided a remarkable synthesis of these two notions. Rossi-Landi (1977: 139) criticised Saussure as ‘he does not appear to possess a theory of linguistic work, the most viable foundation for any theory of linguistic value.’ Moving from Hegel’s anthropogenetic characters of labour and language, Rossi-Landi described a homology between the production of material and linguistic artefacts. According to Rossi-Landi, the internal design of artefacts such as machines can be compared to the internal structure of linguistic artefacts such as verbal expressions and written documents. There exists a ‘syllogism of action’ (Hegel 1816/2010: 732) that innervates both material and linguistic production. For this reason, Rossi-Landi also criticised Bloomfield who gave priority to language over labour when he declared that division of labour is possible thanks to language. Rossi-Landi (1977: 71, note 23) suggested that ‘language in its turn, and with it the whole working of human society, is due to the division of labor’ (Caffoni 2026). Ultimately, Rossi-Landi (1977: 102) recognised the computer as the culmination of the mechanisation of labour and language.

Rossi-Landi’s synthesis remains instructive: it helps in thinking philosophy of language and political economy within a common analytic framework, and it opens a path for understanding how the automation of language—most visibly in LLMs—reconfigures both the production of meaning and the distribution of economic value. A few decades after Rossi-Landi, other Italian economists such as Christian Marazzi (1994) advocated for a linguistic turn in political economy to grasp the transformations of social relations and conflicts in post-Fordism. Marazzi proposed to recognise the role of language as *living labour* and antagonistic dimension before its capture by apparatuses such as communication networks and computers. Marazzi (1994/2011: 35) insisted on fashioning the Turing machine as a ‘linguistic machine’ and a new kind of assembly line centring labour management around language and computational logic. Marazzi, and other Italian philosophers from the tradition of Autonomist Marxism such as Paolo Virno (1990/1996), stressed in particular the economic role of collective knowledge, understood as *general intellect* and *mass intellectuality* (Pasquinelli 2019). Their emphasis on the collective and political dimension of knowledge can be seen as a prophecy of the global AI that came, a few decades later, to measure, mathematise and mechanise the free intellects of post-Fordism. Yet it should be noted that, in advocating for a *linguistic turn* in political economy and debates about labour during the 1990s, nobody could have expected the degree of *linguistic automation* that is taking place today with LLMs. What these positions, and the sociology of labour more generally, lacked was a theory of automation.

## Conclusion

Whereas the epistemology of science and technology has often given emphasis to the organism–machine analogy of early modernity, this essay has tried to shift the focus to another key analogy of late modernity, the language–machine analogy. This analogy was grounded by heuristic technologies such as the telegraph and disciplines such as linguistics, and then evolved into information technologies, digital computers and eventually AI. The two perspectives and polarities of the language–machine analogy—language as machine and machine as language—have crossed the last century, and are part of the same history of formalisation and automation of labour. Language, understood as labour and more precisely as the abstraction of the social division of labour, constitutes the fabric of the machine. Language, however, far from being merely a formalisable and automatable system, remains the space of collective and political agency: one cannot miss, in the mid-2020s, the politicisation of LLMs for ideological propaganda and cultural hegemony. Global cultural wars have been declared along the deep neural networks of AI models. How should critical theory respond to this scale of power that AI systems came to embody? The present essay proposed a historical and comparative epistemology of the paradigms of machine, organism and language to rediscover their role in the genealogy of AI as configurations of labour and society at large. This genealogy will, hopefully, open the way to imagining new forms of political intervention in the constitution of AI models as mirrors of the social order.

## Data Availability

No datasets were generated or analysed during the current study.
